# Role of Endobronchial Ultrasound in the Diagnosis of Bronchogenic Cysts

**DOI:** 10.1155/2011/468237

**Published:** 2011-05-11

**Authors:** Devanand Anantham, Ghee-Chee Phua, Su-Ying Low, Mariko-Siyue Koh

**Affiliations:** Department of Respiratory and Critical Care Medicine, Singapore General Hospital, Outram Road, Singapore 169608

## Abstract

Diagnosis of bronchogenic cysts is possible with computed tomography, but half of all cases present as soft tissue densities. Two such cases are highlighted where asymptomatic bronchogenic cysts that presented as soft tissue masses were evaluated by endobronchial ultrasound (EBUS). After studying the ultrasound image characteristics, the diagnosis was confirmed using EBUS-guided transbronchial needle aspiration (EBUS-TBNA). The first case had ultrasound findings of an anechoic collection, and the aspirate was serous with negative microbiologic cultures. The second was an echogenic collection within a hyperechoic wall. Needle aspirate was purulent and cultured Haemophilus influenza. The diagnosis of a bronchogenic cyst complicated by infection was made, and the lesion was surgically resected. This potential for EBUS in the diagnosis of bronchogenic cysts and in identifying complications such as infection should be considered in the management of such cases.

## 1. Introduction

Bronchogenic cysts are ventral foregut duplication cysts that arise from aberrant embryonic development. They are typically located near the large airways just posterior to the carina. Although many are asymptomatic and discovered incidentally, complications such as infection, airway obstruction, fistulation, vascular compression, and malignant transformation have been reported [1]. 

Bronchogenic cysts are lined with cartilage and pseudostratified columnar epithelium. Noninvasive diagnosis is possible with computed tomography (CT) using typical characteristics of a round, well-circumscribed, unilocular mass [1]. Approximately half of the cysts are homogeneous with a near water density (0–20 Hounsfield Units) that reflects their serous nature. The remainder have attenuation values of soft tissue because of viscid mucoid contents making them indistinguishable from neoplasms [2]. Whilst the additional use of magnetic resonance may help to further characterize these lesions, they can still be confused for malignancies with central necrosis. An exploratory thoracoscopy or thoracotomy may then be necessary to confirm the diagnosis [3]. 

We present two cases where endobronchial ultrasound (EBUS) was performed on patients with asymptomatic bronchogenic cysts. After studying the ultrasonographic images, transbronchial needle aspiration (TBNA) was performed at the same sitting to confirm the diagnosis. Both were outpatient procedures that were completed under local anesthesia and conscious sedation without any complications. The EBUS ultrasound images correlated with TBNA aspirate findings and led to two different management strategies for each of the bronchogenic cysts.

## 2. Case 1

**Figure 1:**
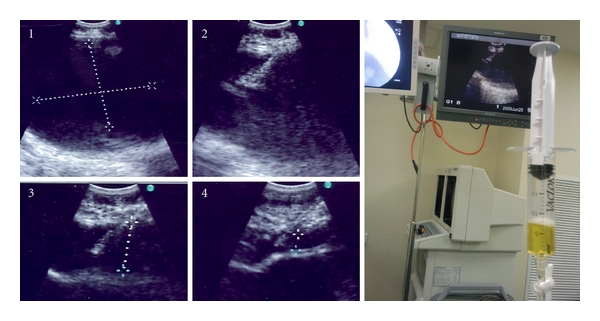
Anechoic cystic lesion getting progressively smaller as serous fluid is being drained via EBUS-TBNA.

A 54-year-old ex-smoker presented with an abnormal chest radiograph. He was asymptomatic had a normal physical examination, and the radiograph was taken as part of a license application. CT scan revealed a well-circumscribed lesion in the right lower paratracheal region abutting the azgos vein. EBUS-TBNA was performed using the 7.5 MHz convex probe bronchoscope (BF-UC260F; Olympus Ltd, Tokyo, Japan). The lesion was identified as a round, anechoic structure and distinguished from surrounding blood vessels using color Doppler (Figure 1). A dedicated 22-gauge needle (NA-202, Olympus Ltd, Tokyo, Japan) was used to puncture and aspirate the lesion under direct visual guidance. To ensure complete drainage of the lesion, the gradual shrinkage of the cyst was observed on ultrasound while 50 milliliters of serous fluid was aspirated (Figure 1). Cytological examination of the aspirate yielded metaplastic squamous cells with negative microbiological cultures. He has remained clinically and radiologically stable on followup for 18 months.

## 3. Case 2

**Figure 2:**
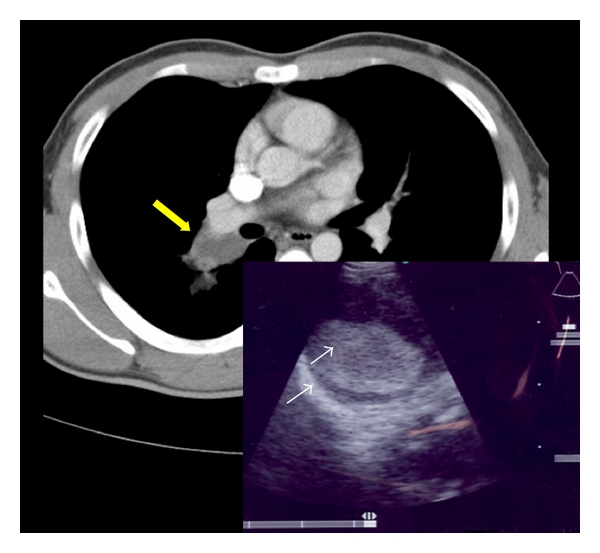
CT scan showing a soft tissue lesion at the right hilum (yellow arrow). Insert image: EBUS image showing a round lesion with an echogenic center and thickened hyperechoic walls (white arrows).

An 18-year-old male presented with an abnormal chest radiograph that was performed before military enlistment. He was also asymptomatic and had a normal physical examination. CT scan revealed a mass in the right hilum. EBUS-TBNA was performed and ultrasound identified a round lesion with an echogenic centre and thickened, hyperechoic walls (Figure 2). Ten milliliters of purulent aspirate was drained. Cytological examination identified numerous neutrophils, occasional squamous cells and an amorphous granular background. Bacterial cultures grew Haemophilus influenza. He was treated with antibiotics and referred for surgical resection. A lobulated mass at the root of the right upper lobe with a cystic cavity was found on right thoracotomy. Histopathological examination revealed a well-defined cystic space lined by inflamed respiratory epithelium consistent with the diagnosis of an intrapulmonary bronchogenic cyst. 

## 4. Discussion

Surgical resection of bronchogenic cysts with complete removal of the secreting mucosal lining is recommended as the therapeutic procedure of choice in cases presenting with complications [1, 4]. However, many cases are, asymptomatic, and the long-term prognosis in such instances is uncertain with a few reports of late complications. The role of preventative surgery remains controversial, and asymptomatic patients have been managed successfully by clinical observation as well.

 Endoscopic aspiration has been attempted via conventional transbronchial needle aspiration, radial (20 MHz) EBUS, endoscopic ultrasound (EUS), and convex probe real-time EBUS-TBNA [5–7]. Needle aspiration is usually associated with a high recurrence rate because the lining of the cyst is not removed. However, this procedure remains a possible palliative measure in patients presenting with airway obstruction who are poor surgical candidates [6]. Furthermore, ultrasound facilitates visualization during aspiration and enables complete aspiration of the cyst that is not always possible when “blind” techniques are utilized. This causes collapse of the cystic space and may facilitate adhesion between the mucosal surfaces lining the cavity, consequently reducing recurrence rates [6, 7]. 

Ultrasound provides excellent delineation between solid and fluid structures. Echogenicity of cystic lesions is related to their cellular content, and echogenicity is by convention described with reference to soft tissue such as lymph nodes or tumors. These structures are grey in color and are termed isoechoic. Darker (hypoechoic) or black (anechoic) images denote either cysts with serous fluid or blood vessels. Echogenic images within cysts identify complicated fluid collections that may contain either frank pus or blood clots [8]. The presence of septations, thickened walls, or floating debris within the cyst may give further radiological clues to an infected cyst [8].

Therefore, EBUS can be used to diagnose bronchogenic cysts that present as soft tissue densities on CT imaging. Uncomplicated cysts can be identified using ultrasound image analysis and subsequently managed by observation. Infected cysts may also be recognized and managed aggressively with early surgical resection. If there is any doubt in the diagnosis, then EBUS-TBNA can be considered and aspirate sent for bacterial cultures and cytological analysis.

Antibiotic prophylaxis is recommended should TBNA be performed because of the risk of introducing infection into the cysts and mediastinum [9]. The TBNA needle may become contaminated with airway commensals as the bronchoscope passes through the oropharyngeal region and these organisms can be transferred during needle aspiration. There are concerns that this infection risk may be especially elevated if the TBNA needle is used in full extension (36 mm). At such depths, the tip of the needle is poorly visualized on ultrasound and may inadvertently puncture surrounding tissues [9].

## 5. Conclusion

EBUS has a potential role when a confident diagnosis of bronchogenic cyst cannot be made by CT. Uncomplicated cysts appear as rounded, anechoic lesions on ultrasound scans. Early identification of complications such as infection is possible because such lesions are echogenic with thickened walls.

##  Authors' Contribution

All authors participated in the research and take responsibility for the paper.
